# Radiation treatment waiting times for breast cancer patients in Manitoba, 2001 and 2005

**DOI:** 10.3747/co.v16i5.298

**Published:** 2009-09

**Authors:** A.L. Cooke, R. Appell, K. Suderman, K. Fradette, S. Latosinsky

**Affiliations:** * CancerCare Manitoba, Winnipeg, MB; † Department of Radiology, University of Manitoba, Winnipeg, MB; ‡ Department of Surgery, University of Manitoba, Winnipeg, MB

**Keywords:** Breast cancer, radiation, wait times

## Abstract

**Introduction:**

Our study examined the wait time from ready-to-treat to radiation therapy for cohorts of breast cancer patients requiring adjuvant radiation therapy in 2001 and in 2005 after the implementation of strategies to reduce wait times for radiation treatment. We also examined the overall time from diagnosis to radiation treatment and whether distance from the cancer treatment centre or month of referral had an effect on wait times.

**Methods:**

This population-based retrospective study looked at representative samples of women newly diagnosed with breast cancer in 2001 and 2005. Patients who required radiation treatment to the breast or chest wall were followed from first contact to the start of radiation treatment.

**Results:**

Time from ready-to-treat to first radiation treatment was significantly reduced for patients in 2005 as compared with 2001, regardless of whether chemotherapy was administered before radiation treatment. Time from diagnosis to radiation treatment was not different by year for those who received radiation only. Time from diagnosis to chemotherapy was significantly longer in 2005. No effect of month of diagnosis on wait times was observed.

**Interpretation:**

A significant improvement in the median wait time from ready-to-treat to first radiation treatment was noted from 2001 to 2005. This improvement may be attributable to measures taken to reduce such waits. However, we observed an increase in the median time from diagnosis to referral and from referral to consultation with medical or radiation oncology (or both), so that the overall time from diagnosis to radiation treatment was not different. Although specific intervals related to radiation treatment delivery were improved, the entire trajectory of breast cancer care experienced by patients needs to be considered.

## 1. INTRODUCTION

Lengthy wait times for radiation therapy (rt) in Canada have been widespread and have received considerable media attention 1–3. During the peak of the crisis in the late 1990s, several jurisdictions, including the province of Manitoba, resorted to sending patients requiring rt to the United States to reduce wait times.

The Manpower and Standards of Care Committee of the Canadian Association of Radiation Oncologists (caro) recommended in 2000 that the interval between referral and consultation not exceed 10 working days (**not** calendar days) and that the interval between consultation and treatment also not exceed 10 working days 4. In 1991, few patients in Canada were treated within these timelines 5. In Canada, patients have sued over the wait time for breast cancer treatment 6. By 2002, the wait time for radiation in Ontario had increased to 7 weeks, and so in 2004, in response to the persistent rt wait time problems, the Cancer Quality Council of Ontario issued a report recommending a 4-point approach to reducing wait times 7.

To reduce wait times for rt in the province of Manitoba, Manitoba Health and CancerCare Manitoba (ccmb) took steps to increase the numbers of radiation therapists by negotiating a competitive labour agreement. Additional medical physicists and radiation oncologists were hired, and a major investment was made in new radiation planning and therapy equipment. The present study was conducted to document whether those measures had affected radiation wait times. Breast cancer was chosen because it is a frequently occurring malignancy with relatively standardized care and recorded events. Sub-analyses investigated whether seasonal variations in radiation wait times could be detected and whether wait times were a function of distance from the cancer treatment centre.

The caro definitions and recommendations deal only with wait times or intervals occurring after referral to radiation oncology; they do not encompass the entire trajectory of care, including care delivered before referral 4. We therefore examined time intervals from first contact and diagnosis through to rt so that any improvement in wait time for rt could be examined in the context of all breast cancer care experienced by the patients.

Our study was approved by the University of Manitoba Health Research Ethics Board and by the Health Information Privacy Committee of Manitoba Health.

## 2. PATIENTS AND METHODS

### 2.1 Study Population

Women with stage 0 to iv breast cancer diagnosed in four representative months (January, June, September, November) in each of 2001 and 2005 were identified from the Manitoba Cancer Registry. Of these women, those who also received local or locoregional adjuvant rt were identified from ccmb health records.

### 2.2 Administrative Data Sources

Under the Cancer Act of the province of Manitoba, ccmb is legally mandated to collect, classify, and maintain accurate comprehensive information on all cancer cases in a population-based central cancer registry, the Manitoba Cancer Registry (mcr). The mcr is annually certified for quality by the North American Association of Central Cancer Registries. All patients in the mcr have a unique cancer registry (cr) number. Multiple sources are used to ensure high levels of case ascertainment. These include physician notifications, pathology and cytology reports, and hospitalization, mortality, and autopsy records. Information about definitive treatment has been captured since 1992 in the mcr, and all breast cancer cases from 1995 onward have been staged.

Data obtained from the mcr included date of diagnosis (determined by the date of the first pathology or cytology report to show carcinoma or carcinoma *in situ* of the breast); age and postal code at diagnosis; TNM information, summary disease stage, and tumour grade; and estrogen receptor, progesterone receptor, and human epidermal growth factor receptor status. Cases in the mcr diagnosed in 2001 are coded using the *International Classification of Diseases, 9th Clinical Modification,* (icd-9-cm) and are staged using the fifth edition of the *AJCC Cancer Staging Manual* from the American Joint Committee on Cancer 8. Cases diagnosed in 2005 are coded using the *International Classification of Diseases, 10th Revision, Canada,* (icd-10-ca) and are staged using the Collaborative Staging System.

All administrative, clinical, and radiation and chemotherapy treatment information is also maintained by ccmb in computerized health records. A paper chart is still maintained for some documents, such as letters of referral and other reports that are not electronic.

Manitoba Health provides comprehensive health care coverage for essentially all residents of the province of Manitoba. Because Manitoba residents are not obliged to pay premiums for this coverage, nonparticipation in the plan is rare, and claims data are relatively complete for the entire population. Manitoba Health maintains computerized health claims databases for most physician services, including all fee-for-service claims made by physicians in the province of Manitoba for all persons registered with the system from 1970 to the present. Each medical claims record includes information on the claiming physician, the service date, and the type of service, which is coded using billing (tariff) codes indicated by Manitoba Health. The accuracy of the Manitoba Health administrative data has been established by the Manitoba Centre for Health Policy for a wide range of clinical disorders 9. Since 1984, every resident of Manitoba has been assigned a unique personal health identification number that can be used to link patient records across administrative health databases. Linkage of the mcr and Manitoba Health medical claims database allows for the creation of a comprehensive record of a patient’s care from first contact through imaging, diagnosis, treatment, and follow-up. To protect confidentiality linkage uses scrambled identification numbers and anonymized versions of the databases.

### 2.3 Wait Time Definitions

The Manitoba Health medical claims file uses separate billing codes for diagnostic and screening mammograms. For asymptomatic cancers detected during routine mammographic screening, first contact is defined as the date of the screening mammogram within 6 months preceding the date of diagnosis. If the patient had a diagnostic mammogram or ultrasound, but not a preceding screening mammogram, the date of first contact is the last physician or surgeon service date immediately preceding the date of the diagnostic mammogram or breast ultrasound within 6 months preceding the date of diagnosis. For women who had no mammogram or ultrasound within 6 months preceding the date of diagnosis, the date of first contact is the physician or surgeon service date immediately preceding the date of diagnosis.

**FIGURE 1:**
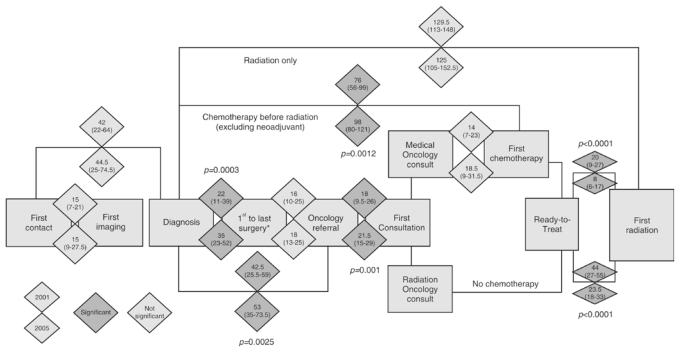
Comparison of 2001 and 2005 wait times for breast cancer patients along the trajectory of care. Intervals between breast cancer events are shown in median days, 2001 compared with 2005. For each pair of diamonds, the top diamond shows the 2001 mean elapsed days (25th–75th percentile), and the bottom diamond shows the 2005 median elapsed days (25th–75th percentile). Significant differences appear in the darker grey shade, and *p* values are adjusted for multiple testing. * Including mastectomy, partial mastectomy, and axillary dissection if carried out at separate times.

Dates of first contact, first imaging (whether screening or diagnostic mammography or breast ultrasound), surgical consultation, and surgery were obtained from the Manitoba Health medical claims database. The date of diagnosis and date of first surgery would be the same (or very close if the pathology report took several days to process) for patients who had an excisional biopsy as their diagnostic procedure. If more than one surgical procedure was required, then the “first surgery” date is defined as the first segmental mastectomy, mastectomy, or axillary node dissection that occurred on or after the diagnosis date; the “last surgery” date is defined as the last segmental mastectomy, mastectomy, or axillary node dissection that occurred on or before the oncology referral date (Figure 1).

Dates of referral to oncology, dates of medical and radiation oncology consultation, and chemotherapy start dates were obtained from ccmb health records. The date of oncology referral is defined as the date on which a referral for medical or radiation oncology assessment (or both) was received at ccmb or received by a community-based medical oncologist before referral to ccmb for radiation oncology consultation. Referral dates for 34 patients who were seen by a community-based medical oncologist before being referred to ccmb for radiation oncology consultation were not available. For these patients, a proxy referral date was assigned using the date of the pathology report of the last surgery before the community-based medical oncology consultation. For patients who received neoadjuvant chemotherapy following a diagnostic procedure, the date of the pathology or cytology report of the diagnostic procedure was used as a proxy for the referral date. The date of consultation was the date on which the first oncologist (whether medical or radiation) saw the patient.

Because patients who have chemotherapy first, followed by rt, have an interval before rt that is not waiting *per se,* another date, “ready-to-treat” was defined. “Ready-to-treat” is defined as the date on which a decision is made by the radiation oncologist and the patient that rt is indicated and that the patient is medically ready and willing to begin treatment. This definition is in keeping with the caro 2000 recommendations for multimodality treatment 4. The ready-to-treat date was determined by the wait list coordinator from ccmb health records. For patients who received chemotherapy, the ready-to-treat date was 4 weeks after the last dose of scheduled and administered intravenous cytotoxic therapy. For patients who received rt only, “ready-to-treat” would usually be the date of the rt requisition, which is usually also the date of the radiation oncology consultation. Patients will occasionally ask for rt deferrals for personal reasons, which would constitute the balance of the cases, and in these cases, “ready-to-treat” would be defined as the date on which the patient returns and agrees to proceed. Distance from the cancer treatment centre was determined by the postal code of the patient. Patients were classified as urban (within Winnipeg), near-urban (within 100 km of Winnipeg), and rural (more than 100 km from Winnipeg).

Sequential and exclusive time intervals and composite time intervals measured in elapsed calendar days were calculated as indicated in Figure 1.

### 2.4 Statistical Analyses

The distribution of a time interval may show skew in this type of analysis. For that reason, comparisons used the median two-sample test (two-sided). Corrections were made for multiple testing, where appropriate. All tabulations and statistical analyses were performed using SAS version 9.1 (SAS Institute, Cary, NC, U.S.A.).

## 3. RESULTS

From the mcr, we identified 641 cases of breast carcinoma that were diagnosed in the months of interest in 2001 and 2005. Three cases with diagnoses of sarcoma, leiomyosarcoma, and phyllodes tumour were removed from the study. Radiation treatment to the breast or chest wall was prescribed in 332 cases (330 patients, 2 with bilateral breast cancer). The patients in those cases are the subjects of the present study.

Table I shows the patient tumour and treatment characteristics by year, together with chi-square test results to determine whether those characteristics varied by year of diagnosis. Only patients who received rt with or without chemotherapy or hormonal therapy (or both) are included. The increase in newly diagnosed patients from 2001 to 2005 (156 vs. 176) is consistent with the expected 2%–3% annual increase in the province of Manitoba 10. No significant differences were seen between the 2001 and 2005 cohorts, with the exception of an increased use of sentinel node biopsy in 2005. A small, nonsignificant, increase in accelerated hypofractionated whole-breast radiation is noted. No effect of month of referral on wait times could be detected. All patients in a given year were pooled for further analysis.

Figure 1 outlines the flow of patients through breast cancer care events from first contact to the beginning of rt. A large and significant improvement in the median wait time from ready-to-treat to rt was noted regardless of whether chemotherapy was given before rt. For those who received chemotherapy, the median time from ready-to-treat to rt was reduced to 8 days from 20 days (*p* < 0.0001). For those not receiving chemotherapy, the median time from ready-to- treat to rt was reduced to 23.5 days from 44 days (*p* < 0.0001). In both 2001 and 2005, patients who received chemotherapy tended to have a lesser wait time for rt after ready-to-treat than did patients who did not receive chemotherapy.

Because the first contact date is derived using an algorithm based on administrative data and may include a proxy date, it is considered less certain, and we have used date of diagnosis as a starting point for our composite intervals. The care path splits into those who did and who did not receive chemotherapy. For patients who received chemotherapy first, the median wait time from diagnosis to first chemotherapy significantly increased to 98 days from 76 days (*p* = 0.0012). However, median wait time from medical oncology consult to first chemotherapy did not increase significantly (14 days vs. 18.5 days, *p* = 0.16), suggesting that the wait time from diagnosis to chemotherapy was increased by events before the medical oncology consultation. The time waited by patients from referral to oncology consultation also showed a slight but significant increase for the wait to see a medical or radiation oncologist to 21.5 days from 18.0 days (*p* = 0.001).

Overall, the median wait time from diagnosis to rt for those receiving radiation only was not different from 2001 to 2005 at 129.5 days compared with 125 median days respectively (*p* = 0.55).

The time from diagnosis to first surgery was significantly increased for the 2005 cohort, at 22 days compared with 35 days (*p* = 0.0003), as was the time from surgical consult to first surgery, 15 days compared with 20 days (*p* = 0.0022, data not shown). The median wait time from diagnosis to oncology referral, which is a composite measure of all activities leading up to referral, including additional imaging, surgical consultation, surgery or surgeries, and pathology turnaround times significantly increased from 2001 to 2005, at 42.5 days compared with 53 days (*p* = 0.0025).

Distance from the cancer centre as identified using postal code at diagnosis was examined to determine if a correlation with wait time intervals was evident (Table II). A significant difference in median wait time from diagnosis to referral was observed for the three distance categories in 2001 (*p* = 0.0093), but no difference was found in 2005. Median wait time from ready-to-treat to rt was not affected by distance from the cancer centre in either year (data not shown).

## 4. DISCUSSION

In this study of patients receiving breast or chest wall rt for newly diagnosed breast cancer at ccmb, the median wait time from ready-to-treat to rt significantly improved from 2001 to 2005. A decrease of 16 days for patients receiving chemotherapy first and a decrease of 20.5 days for those receiving rt alone were observed, suggesting that the investments made by Manitoba Health and ccmb in staffing, treatment hardware, and planning software were successful in reducing wait times for rt. However, no improvement in the overall median time from diagnosis to rt was observed for those receiving radiation only.

A significant worsening in the time from diagnosis to chemotherapy was noted, but no significant difference in the wait time from medical oncology consultation to chemotherapy was seen, suggesting that the increased overall time to chemotherapy was a result of events before consultation.

In both years, the time from ready-to-treat to rt was shorter for patients who received chemotherapy before rt than for those who did not. This finding likely reflects the advance notice given to the rt program for patients receiving chemotherapy first; it is consistent with work in Quebec, which demonstrated that delays to rt are more prevalent among patients who do not receive chemotherapy before radiation, and with data from Ontario, which showed that patients who did not receive chemotherapy experienced the longest waits for rt 11,12.

In the present study, improvement in the timeliness of rt delivery for those who were treated with rt only was negated by a worsening of the median times from diagnosis to surgery and from referral to oncology consultation such that the overall elapsed time experienced by the patients remained the same. This finding suggests that the entire care experience of patients needs to be considered and that an improvement in one wait list that is only a part of a sequence of wait lists does not necessarily result in an overall benefit, an observation that is consistent with research from Nova Scotia that demonstrated the importance of evaluating time intervals along the entire trajectory of care so as to better understand the location and magnitude of changes in wait times 13,14.

Despite major improvements by 2005, neither the 75th percentile nor the median wait time from ready-to-treat to rt met caro guidelines 4 except for patients who needed chemotherapy first. Recent experience at ccmb in 2008 indicates considerable further improvement since 2005 in rt wait times, which are now approaching caro recommendations (CancerCare Manitoba. Month End Wait List Summary. Internal communication).

The worsening of the median wait time before oncology referral appears mostly to be a result of time from diagnosis to first surgery. This interval is a composite of the turnaround time of the biopsy report; the availability of a breast cancer surgeon, anesthetist, and operating room time or hospital bed availability for patients who require an inpatient recovery period; and the speed with which participants in the system respond. Using administrative data, this study cannot address the contribution of each of these subintervals in individual patients. Although the intervals cannot be directly compared because of differences in methodology, an overall worsening of the time intervals before radiation or medical oncology referral was similarly reported in Nova Scotia, where it was shown that the intervals comprising biopsy-to-surgery and surgery-to-referral both experienced prolongation between two cohorts (1999–2000 vs. 2003–2004) 13. In the present study, the increase in time from referral to consultation also contributed to the overall wait time and did not meet caro guidelines 4 in either year. Some of the delay was a result of long wait lists for medical oncology consultation experienced at ccmb during the years of the study.

We did not analyze all cases diagnosed in 2001 and 2005. The months sampled deliberately included times of the year during which wait times might be expected to fluctuate. Our data did not reveal any effect of month of diagnosis on wait times.

In a large jurisdiction such as Manitoba, there is concern that rural patients may be disadvantaged in access to care. In 2001, differences for wait times from diagnosis to referral were noted for the three geographic patient groups (Table II). By 2005, however, there was no evidence that any patient group had any advantage in wait times.

The oncologic consequences of delays in commencing adjuvant radiation treatment post lumpectomy have been examined. In a study conducted across Quebec, breast cancer patients who waited more than 12 weeks after surgery for rt had a higher rate of local failure (hazard ratio: 1.75; 95% confidence interval: 1.00 to 3.08) after multivariate adjustment, including the effect of systemic therapy 15. No difference in survival was observed. In a single-institution study, also from Quebec, the time to breast rt was associated with an increase in local recurrence in univariate analysis, but the effect became equivocal in multivariate analysis 16. A study conducted in Ontario could not find an effect of time to rt on local control in a stepwise multivariate Cox regression model 17,18. Theoretic models of tumour control probability predict that delays in initiating treatment will have an adverse effect on local control, especially for rapidly growing tumours 19.

Regardless of the oncologic effect of a delay in initiating breast rt, there are psychological and social consequences for patients, families, and treating professionals who must manage a rt wait list 20,21.

## 5. CONCLUSIONS

Investments by Manitoba Health and ccmb in staffing, treatment hardware, and planning software were associated with significant reductions in median wait times for breast rt from 2001 to 2005 as measured from ready-to-treat to commencement of rt, but still did not meet caro guidelines. Gains in rt wait times between 2001 and 2005 for those receiving radiation alone were equal in magnitude to increases in wait times that developed elsewhere in the sequence of care steps, so that the overall time from diagnosis to rt was not significantly improved. The entire trajectory of patient care needs to be considered and not just the time spent on the rt wait list.

## Figures and Tables

**TABLE I:** Comparison of the 2001 and 2005 cohorts

Characteristic	2001		2005		*p* Value^a^
	(*n*)	(%)	(*n*)	(%)	
Patients	156		176		—
Diagnosis month					
Jan	47	30.1	47	26.7	ns
Jun	33	21.2	45	25.6	
Sep	36	23.1	38	21.6	
Nov	40	25.6	46	26.1	
Stage					
0	20	12.8	28	15.91	ns
i	69	44.2	74	42.05	
ii	54	34.6	48	27.27	
iii/iv	13	8.3	26	14.77	
Grade					
i	30	19.2	34	19.32	ns
ii	75	48.1	78	44.32	
iii	42	26.9	53	30.11	
Unknown	9	5.8	11	6.25	
Age at diagnosis					
≤49 years	43	27.56	31	17.61	ns
50–69 years	78	50	114	64.77	
≥70 years	35	22.44	31	17.61	
Type of surgery					
Lumpectomy	127	82.5	134	77	ns
Mastectomy	27	17.5	40	23	
Sentinel node biopsy					
Yes	18	11.5	102	58	<0.0001
No	138	88.5	74	42	
Nodal status					
Positive	54	39.1	55	37.2	ns
Negative	74	53.6	86	58.1	
Unknown	10	7.2	7	4.7	
Estrogen receptor status					
Positive	113	72.4	132	75	ns
Negative	37	23.7	39	22.2	
Unknown	6	3.8	5	2.8	
Progesterone receptor status					
Positive	95	60.9	120	68.2	ns
Negative	54	34.6	51	29	
Unknown	7	4.5	5	2.8	
Chemotherapy					
Yes	73	46.8	76	43.2	ns
No	83	53.2	100	56.8	
Radiation therapy					
4000–4999 cGy	36	23.2	50	28.9	ns
5000+ cGy	119	76.8	123	71.1	

a By chi-square test with Bonferroni correction.

**TABLE II:** Wait time from diagnosis to referral by year and postal code of residence

Postal code	Patients (*n*)		Median days	
	2001	2005	(25th–75th percentile)	
			2001	2005
Urban	109	117	37 (20–54)	53 (37–74)
Near-urban	24	30	49 (35–64.5)	52.5 (32–680)
Rural	23	29	64 (40–72)	55 (31–76)
*p* Value^a^			0.0093	ns

a By chi-square test.
